# Delayed Onset of Symptoms and Atovaquone-Proguanil Chemoprophylaxis Breakthrough by *Plasmodium malariae* in the Absence of Mutation at Codon 268 of *pmcytb*

**DOI:** 10.1371/journal.pntd.0004068

**Published:** 2015-10-20

**Authors:** Beatrix Huei-Yi Teo, Paul Lansdell, Valerie Smith, Marie Blaze, Debbie Nolder, Khalid B. Beshir, Peter L. Chiodini, Jun Cao, Anna Färnert, Colin J. Sutherland

**Affiliations:** 1 Department of Immunology & Infection, Faculty of Infectious & Tropical Diseases, London School of Hygiene & Tropical Medicine, London, United Kingdom; 2 Public Health England Malaria Reference Laboratory, London School of Hygiene & Tropical Medicine, London, United Kingdom; 3 Department of Clinical Parasitology, Hospital for Tropical Diseases, London, United Kingdom; 4 Jiangsu Institute of Parasitic Diseases, Wuxi, China; 5 Dept Medicine Solna, Karolinska Institutet, Stockholm, Sweden; 6 Department of Infectious Diseases, Karolinska University Hospital, Stockholm, Sweden; Institut Pasteur, FRANCE

## Abstract

*Plasmodium malariae* is widely distributed across the tropics, causing symptomatic malaria in humans with a 72-hour fever periodicity, and may present after latency periods lasting up to many decades. Delayed occurrence of symptoms is observed in humans using chemoprophylaxis, or patients having received therapies targeting *P*. *falciparum* intraerythrocytic asexual stages, but few investigators have addressed the biological basis of the ability of *P*. *malariae* to persist in the human host. To investigate these interesting features of *P*. *malariae* epidemiology, we assembled, here, an extensive case series of *P*. *malariae* malaria patients presenting in non-endemic China, Sweden, and the UK who returned from travel in endemic countries, mainly in Africa. Out of 378 evaluable *P*. *malariae* cases, 100 (26.2%) reported using at least partial chemoprophylaxis, resembling the pattern seen with the relapsing parasites *P*. *ovale* spp. and *P*. *vivax*. In contrast, for only 7.5% of imported UK cases of non-relapsing *P*. *falciparum* was any chemoprophylaxis use reported. Genotyping of parasites from six patients reporting use of atovaquone-proguanil chemoprophylaxis did not reveal mutations at codon 268 of the cytb locus of the *P*. *malariae* mitochondrial genome. While travellers with *P*. *malariae* malaria are significantly more likely to report prophylaxis use during endemic country travel than are those with *P*. *falciparum* infections, atovaquone-proguanil prophylaxis breakthrough was not associated with *pmcytb* mutations. These preliminary studies, together with consistent observations of the remarkable longevity of *P*. *malariae*, lead us to propose re-examination of the dogma that this species is not a relapsing parasite. Further studies are needed to investigate our favoured hypothesis, namely that *P*. *malariae* can initiate a latent hypnozoite developmental programme in the human hepatocyte: if validated this will explain the consistent observations of remarkable longevity of parasitism, even in the presence of antimalarial prophylaxis or treatment.

## Author Summary

*Plasmodium malariae* is a widely distributed parasite infecting humans across the tropics, and causing symptomatic malaria with a 72-hour fever periodicity. It is rarely studied. This parasite has the notable ability to recur after many decades of apparent absence. Further, people who have received drug treatment for other malaria infections, or to protect themselves when travelling, sometimes develop malaria illness due to *P*. *malariae* resurgence months or years later. We set out to understand the ability of this largely ignored parasite to persist in the human host. To do this we studied 359 *P*. *malariae* malaria patients in non-endemic China, Sweden, and the UK following endemic country travel, mainly in Africa. These patients were much more likely to report protective use of malaria drugs while travelling than were patients with *P*. *falciparum* infection. We tested for drug resistance, but did not find any evidence that this played a part in parasite survival in these travelers. We propose that *P*. *malariae* may have a relapsing form in the liver, which permits emergence of parasites into the blood months, years or decades after initial infection from a mosquito bite.

## Introduction

Human malaria is caused by six species of protozoan parasite: *Plasmodium falciparum*, *P*. *knowlesi*, *P*. *malariae*, *P*. *ovale curtisi*, *P*. *ovale wallikeri* and *P*. *vivax* [1]. According to the World Malaria Report 2014 [2], it is estimated that 198 million cases (uncertainty interval, 124–283 million) and 584 000 malaria deaths (uncertainty interval, 367 000–755 000) occurred in 2014. Between 1994 and 2014, imported malaria was diagnosed in 38,198 travellers returning to the United Kingdom [3,4]. The least common parasite species reported to the PHE Malaria Reference Laboratory (MRL) during this period were *P*. *knowlesi* (1 case), and *P*. *malariae*, responsible for 688 cases, 1.8% of the total overall, although in both 2013 and 2014 *P*. *malariae* infections accounted for 2.6% of all imported malaria [3,4]. Little attention has been paid to the latter species, and no particular chemoprophylaxis recommendations have been developed for *P*. *malariae*, the general assumption being that infection with this species will be prevented by any prescribed regimen that is effective against the more common *P*. *falciparum* or *P*. *vivax*. Despite being widely distributed and present in all malaria endemic zones, only a single case of *P*. *malariae* infection, in Costa Rica, is mentioned in the most recent World Malaria Report [2].

The existing literature presents a conflicting picture of the drug responses of *P*. *malariae*. Whereas an acceptable level of efficacy of most antimalarial drugs against *P*. *malariae* has been reported in both field and clinic [5–7], recent data from Ghana and Uganda suggest that sub-microscopic persistence of *P*. *malariae* infections after ACT treatment is not uncommon [8,9]. Further, there are sporadic case reports of recrudescent *P*. *malariae* parasitaemia in travellers treated weeks or months prior for imported *P*. *falciparum* malaria [10,11], and renowned instances of symptomatic *P*. *malariae* malaria in individuals with no reported exposure to infected *Anopheles* mosquitoes for several decades [12–14]. These observations are difficult to explain if, as current dogma would suggest, *P*. *malariae* does not have a latent liver-stage hypnozoite analogous to that well-described for *P*. *vivax* and the monkey parasite *P*. *cynomolgi* [15–16].

Among the more effective regimens for chemoprophylaxis in travellers, atovaquone as a fixed dose combination with proguanil (AP) is particularly recommended in areas where *P*. *falciparum* is prevalent [17]. Although AP is also considered effective in both treatment and chemoprophylaxis for the erythrocytic stages of *P*. *vivax*, *P*. *ovale* spp., and *P*. *malariae* [18,19], we have recently shown that the two parasite species that cause ovale malaria, *P*. *ovale curtisi* and *P*. *ovale wallikeri*, can occur despite patients reporting good adherence to AP chemoprophylaxis during endemic country travel [20]. Recrudescence of *P*. *falciparum* three or more weeks after treatment does occur in a small proportion of AP-treated acute malaria patients [21–23]. Sequencing of the mitochondrial *pfcytb* locus of these recrudescent parasites has confirmed that a single nucleotide polymorphism at codon 268 (Tyr to Asn, Ser or Cys) is associated with treatment failure *in vivo* [21–23]. Despite these documented therapeutic failures, there are only two cases of fully compliant AP chemoprophylaxis breakthrough of *P*. *falciparum* infection documented in the literature, and this regimen has an estimated prophylactic efficacy against falciparum malaria of greater than 99.9% [24]. Although AP may offer some protection against *P*. *vivax* liver stages [19], one report describes a *P*. *vivax*-infected patient who experienced both chemoprophylaxis breakthrough and treatment failure with AP, the relapse occurring five months after the initial episode in the absence of *pvcytb* mutations [25]. Curiously, despite the apparent lack of hypnozoites in this species, there are also reports of *P*. *malariae* surviving treatment and chemoprophylaxis with a number of regimens including AP [11, 24, 26]. However, *cytb* mutations have not previously been evaluated in *P*. *malariae*, so it is unclear whether this phenomenon represents an acquired resistance mechanism, or an intrinsic biological characteristic of this parasite.

In this study, estimates of the latency period of imported *P*. *malariae* infections were derived from data compiled by specialist centres in China and Sweden and from the UK MRL database. Cases of *P*. *malariae* infection in travelers reporting use of AP for chemoprophylaxis, and for whom frozen blood samples were available for DNA analysis, were identified in the UK MRL database between 2007 and 2010, and sequences at codon 268 of *pmcytb* elucidated in each of these putative breakthrough infections. Our findings are discussed with reference to published examples of long-term persistence of infections with this parasite species, and propose reconsideration of the possibility that latent liver-stage hypnozoites do occur in *P*. *malariae*.

## Methods

### Study population

Anonymised clinical and epidemiological data on cases of *P*. *malariae* malaria reported to the PHE MRL, London, from 1991 to 2010, cases of *P*. *malariae* malaria seen at the Karolinska University Hospital, Stockholm, Sweden, between 1997 and 2013 and cases of *P*. *malariae* infection diagnosed in Jiangsu Province and reported to the Institute for Parasitic Diseases, Wuxi, China, were available. For a subset of the London series, DNA samples were provided for *pmcytb* gene sequencing. All cases in each site were identified by expert microscopists working at Reference Laboratory standard. PCR confirmation was also carried out for all cases from China, five of those from Sweden and all six UK cases subjected to *pmcytb* sequencing.

### Sample identification

The sample set for *pmcytb* analysis was identified among entries in the database collected by the PHE MRL at the London School of Hygiene and Tropical Medicine (LSHTM). Patients were selected who satisfied all three of the following criteria:

1. reported use of AP chemoprophylaxis whilst travelling abroad;
2. diagnosis of slide-positive *P*. *malariae* infection confirmed by the MRL;
3. availability of a blood sample or purified DNA in the MRL sample archive.

Sixteen samples were identified which fitted criteria 1 and 2, for six of which a blood sample was available. Drug resistance testing was performed under the surveillance remit of the MRL. All patient identifiers have been removed.

### DNA extraction and PCR for detection of codon 268 mutations in cytochrome b

Parasite DNA was extracted from patient blood samples using the QIAamp DNA Blood Mini Kit (QIAGEN, Hilden, Germany) according to the manufacturer’s instructions. Primers were designed specifically for this study, based on *P*. *malariae* cytochrome b (*pmcytb*; NCBI accession number: AB354570.1). Primer sequences were as follows:

Pmcytb1 5’-TGTTTTACCATGGGGACAAAT-3’

Pmcytb2 5’-CACCAATACAAATTGTTCCAGA-3’

Pmcytb3 5’-TGTGGTAATTGACATCCAATCC-3’

Sequencing templates were amplified using a hemi-nested protocol which generated a 635bp nest 1 product (Pmcytb1 + Pmcytb3) and a 253bp nest 2 product (Pmcytb2 + Pmcytb3) encompassing codons 255 to 337 of the *pmcytb* locus. The presence of mutations at codon 268 was investigated by direct sequencing of PCR amplicons.

For the primary amplification reaction, a 25μL mix that contained 1X KCl PCR buffer (Bioline, UK), 15mM MgCl2, 0.2mM deoxyribonucleotide triphosphates (Bioline, UK), 0.25nM each of primers Pmcytb1 and Pmcytb3 (Eurofins, Germany), 0.2μL Taq polymerase (Bioline, UK), and 2μL of extracted parasite DNA was initially heated at 92°C for 3 minutes, then cycled at 92°C for 30 seconds, 55°C for 50 seconds, and 68°C for 90 seconds for 35 cycles, with a final extension at 68°C for 5 minutes. 1μL of PCR product was then added to 19.3μL of nuclease-free water, 0.5μL Pmcytb2 and Pmcytb3 each, and identical volumes of dNTPs, Taq polymerase, and PCR buffer as described in the first amplification reaction to make a total mix of 25μL. 30 further cycles of PCR were applied, as above. PCR products were sequenced using the ABI Prism BigDye Terminator kit (Applied Biosystems, Warrington, UK) according to the manufacturer’s instructions, primed by Pmcytb2 and Pmcytb3. Sequencing data was visualized and analyzed using Chromas software (Technelysium, Australia).

### Statistical analysis

Continuous data (number of days delay to appearance of symptoms) were not normally distributed, and comparisons between categories were therefore performed using the non-parametric Wilcoxon’s rank-sum test. Categorical data were analysed in 2 x 2 tables to provide odds ratios (OR), and statistical significance was estimated from the χ^2^ distribution. Statistical analysis was performed in STATA 12 (StataCorp, USA). All data were de-linked from personal identifiers.

## Results

### Analysis of latency in imported *P*. *malariae* cases from three countries

Two parameter, time elapsed from arrival in a non-endemic setting to symptomatic presentation for diagnosis, and the delay to onset of symptoms, were explored among 638 cases of *P*. *malariae* infection. These comprised:

- 6 travellers diagnosed as imported cases of *P*. *malariae* malaria in various hospitals, and confirmed by the Jiangsu Institute of Parasitic Diseases (JIPD), in Jiangsu province, China between 2012 and 2013
- 21 cases of imported *P*. *malariae* malaria identified at Karolinska University Hospital, Stockholm, Sweden, between 1997 and 2013
- 611 cases of imported *P*. *malariae* malaria presenting to UK hospitals between 1991 and 2010 and reported to the UK MRL.

For many individuals, insufficient data were available to estimate either or both parameters of interest. Table 1 presents a summary of evaluable data from all three sources.

**Table 1:** Summary of delay from arrival in non-endemic region to onset of symptoms and to date of diagnosis for 326 cases of imported *P*. *malariae* infection.

Patient origin	Total cases	Travel destinations (frequency)	Median delay from arrival to onset of symptoms (IQR) [range]	Median delay from arrival to diagnosis (IQR) [range]
China	6	Equatorial Guinea (3)	20 days	27.5 days
		Angola (1)	(5–37)	(13–37)
		Liberia (1)	[2–126]	[9–127]
		Nigeria (1)	N = 6	N = 6
Sweden	21	Uganda (5)	19 days	31 days
		The Gambia (4)	(13–47)	(20.5–59.5)
		Kenya (3)	[0–70]	[3–84]
		Ghana (2)	N = 20	N = 20
		5 other African origins		
United Kingdom	611	Nigeria (150)	24 days	31 days
		Uganda (64)	(9–50)	(16–66)
		Ghana (61)	[-5–1123]	[0–1758]
		Kenya (46)	N = 248	N = 333*
		Congo (16)		
		Cameroon (15)		
		Malawi (13)		
		Sierra Leone (13)		
		Gambia (12)		
		Tanzania (12)		
		Ivory Coast (8)		
		Mozambique (7)		
		Zambia (4)		
		Zimbabwe (4)		
		Sudan (3)		
		Liberia (2)		
		Madagascar (2)		
		Senegal (2)		
		South Africa (2)		
		Togo (2)		
		*10 single African origins;*		
		*55 unspecified “Africa”;*		
		*Non-African origin*:		
		Brunei (1)		
		French Guiana (1)		
		Guyana (1)		
		Malaysia (1)		
		New Guinea (1)		
		Pakistan (1)		
		Yemen (1)		

* Date of diagnosis was more reliably collected than date of onset of symptoms in the UK dataset.

**Fig 1:**
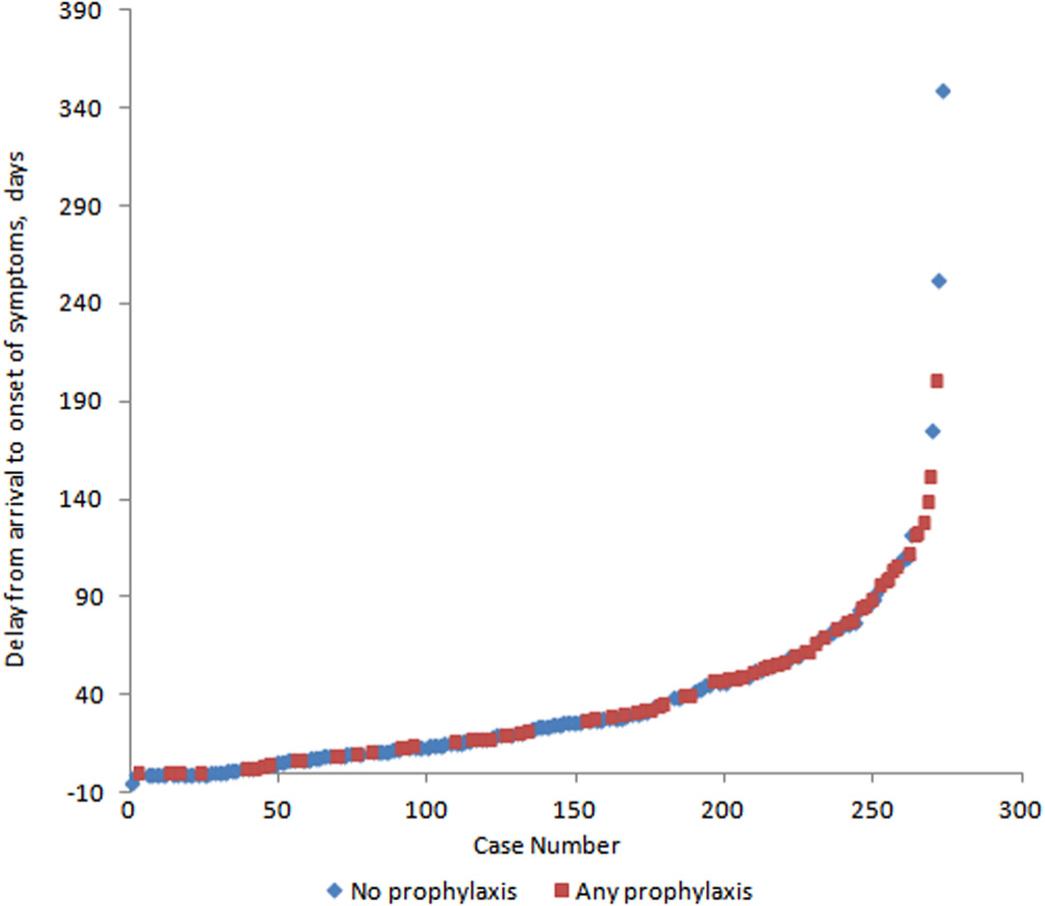
Delay to onset of symptoms in 273 *P*. *malariae* patients presenting with imported malaria in China, Sweden and the UK. Blue markers denote those reporting no chemoprohylaxis use. One outlier from the UK dataset (delay to onset: 1123 days) was excluded.

Reported use of chemoprophylaxis prior to diagnosis with *P*. *malariae* malaria was tested as a likely modulator of delay to onset of symptoms. Patients reporting no chemoprophylaxis while travelling in endemic countries experienced a geometric mean delay of 20.0 days (95% CI 16.5–24.2; N = 146) between arrival in the non-endemic country and onset of symptoms, compared to a geometric mean delay of 36.1 days (95% CI 28.8–45.3; N = 72) in those reporting any chemoprophylaxis use (Wilcoxon’s ranksum test z = -4.106; P < 0.001). Fig 1 presents the distribution of delay to onset of symptoms in these two groups of patients.

Among 77 patients reporting use of a particular chemoprophylaxis regimen, and for whom date of onset of symptoms was also available, the three most frequently used drugs were mefloquine (N = 29), chloroquine (with or without proguanil; N = 19) and AP (N = 17). Of these, mefloquine generated the greatest increase in geometric mean delay to symptoms (53.9 days; 95% CI 38.3–75.7), followed by AP (34.3 days; 95% CI 20.3–58.1). In total, 26.5% of the 378 *P*. *malariae* malaria cases for which data were available reported chemoprophylaxis use, an estimate very similar to that for 4,133 cases of the relapsing parasite *P*. *vivax* (Table 2; P = 0.2315). In contrast, only 7.7% of a data sample of 2, 814 *P*. *falciparum* malaria cases entered in the UK MRL database between 1991 and 2010 reported chemoprophylaxis use (P < 0.0001) [20].

**Table 2:** Proportion of imported malaria cases reporting chemoprophylaxis use, by species.

	Px not used	Px used	Total	OR *P*. *malariae* vs other; (95% CI)	*P*
*P*. *malariae* (this study)	278	100	378	-	-
	73.5%	26.5%			
*P*. *falciparum* *	2,597	217	2,814	4.30; (3.26–5.66)	**< 0.0001**
	92.3%	7.7%			
*P*. *ovale* spp.*	667	378	1,045	0.634; (0.48–0.83)	**0.0006**
	63.8%	36.2%			
*P*. *vivax* *	3,153	980	4,133	1.16; (0.901–1.48)	0.232
	76.3%	23.7%			
**Total**	6,695	1,675	8,370	-	-
	80.0%	20.0%			

Px–chemoprophylaxis;

Px used–chemoprophylaxis use reported, any regimen

OR–odds ratio for not using chemoprophylaxis compared to *P*. *malariae* data

* UK MRL data 1991–2010 [20].

### Role of *pmcytb* mutations in AP chemoprophylaxis breakthrough

**Fig 2:**
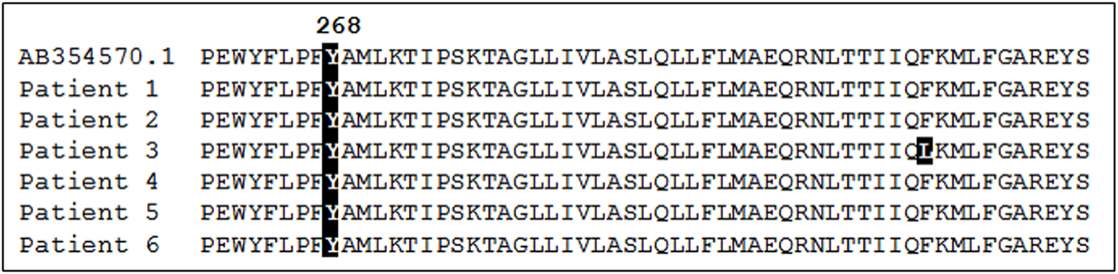
Gene sequencing results of second amplification products with previously published *P*. *malariae* cytochrome b sequence as reference. Y268 is clearly indicated by shading, showing the wild-type Y268 residue is present in all isolates. One residue differing from the reference sequence in patient 3 is highlighted.

DNA sequence data were successfully obtained for isolates from six patients with recorded use of AP chemoprophylaxis prior to onset of malaria. These data were aligned with published *P*. *malariae* cytochrome b sequence (NCBI accession number: AB354570.1) isolated from a human case (Fig 2). No mutations were present at position 268 in any patient isolate. One other non-synonymous difference from the *pmcytb* reference sequence was identified. Patient 3, a 20 year old Caucasian British male with a history of recent travel to Uganda, exhibited a single Phe306Leu mutation, previously described from a *P*. *malariae* infection in one wild chimpanzee, *Pan troglodytes verus*, from Côte d’Ivoire (Genbank Accession: ADP68607.1) [27].

## Discussion

This study of imported cases of *P*. *malariae* malaria evaluated the time elapsed between post-travel arrival in China, Sweden or the UK and clinical presentation and diagnosis of malaria as a proxy measure of latency. A significant relationship between use of chemoprophylaxis and delayed presentation was observed, although some late presentations with *P*. *malariae* malaria were also seen in patients with no history of chemoprophylaxis use during travel. Out of 359 evaluable *P*. *malariae* cases in this study, 26.5% reported using at least partial chemoprophylaxis, closely resembling the patterns seen with the relapsing parasites *P*. *ovale curtisi*, *P*. *ovale wallikeri* and *P*. *vivax*, whereas for 2,814 imported cases infected with the non-relapsing parasite *P*. *falciparum*, only 7.5% reported chemoprophylaxis use (Table 2) [20]. No evidence was found that mutations in the parasite locus *pmcytb* were the cause of breakthrough in 6 travelers who reported using atovaquone-proguanil chemoprophylaxis while at risk of malaria infection. This suggests an intrinsic biological property of this parasite species permits evasion of chemoprophylaxis in a small number of travelers. Our favoured explanation is that the human malaria parasite *P*. *malariae* is also a relapsing parasite, with the ability to form latent hypnozoite stages in the host liver, and that these can initiate new intra-erythrocytic asexual replication months or years after the initial infective mosquito bite. However, we cannot rule out the possibility that *P*. *malariae* may have a hitherto undescribed persistence strategy, such as the generation of metabolically arrested blood-stage forms, or an alternative extra-erythrocytic cycle in a tissue other than liver, which permits evasion of chemoprophylaxis.

We found evidence that the delay from leaving the endemic area to onset of symptoms in imported malaria cases caused by *P*. *malariae* has a wide range, from 0 to 1123 days, and that chemoprophylaxis use makes it significantly more likely that this delay would be extended. In fact, over a quarter of our cases reported chemoprophylaxis use, such that in the UK dataset a clinical case of *P*. *malariae* malaria is 4 times more likely to occur in a traveller who used chemoprophylaxis than is a *P*. *falciparum*–infected case (Table 2; P < 0.0001), but is no more or less likely to do so than cases of vivax malaria. Thus the epidemiology of imported *P*. *malariae* infections mirrors that of the relapsing parasite *P*. *vivax*, but is significantly different from that of the non-relapsing parasite *P*. *falciparum*. These data are consistent with the findings of Schwartz *et al*. [26] who, in a series of 2,822 imported malaria cases from the USA in the 1990s (when chloroquine was typically deployed) found that 13.6% of *P*. *falciparum* cases reported compliant use of prophylaxis, compared to 31.5%, 42.9% and 29.0% for *P*. *malariae*, *P*. *ovale* spp. and *P*. *vivax*, respectively.

In our series of 6 patients with *P*. *malariae* malaria who reported atovaquone-proguanil chemoprophylaxis use, the absence of *pmcytb* mutations at codon 268 provides some evidence that this is an intrinsic property of the parasite rather than an acquired genetic resistance mechanism. Conversely, these observations may reflect drug malabsorption (which we were not able to investigate), or mutations at other positions of *pmcytb*, as were reported in experimentally drug-pressured atovaquone-resistant *P*. *yoelii* parasite lines, where codons other than position 268 of the *pycytb* locus were found to be substituted with novel amino acids [28]. Further sequencing could explore this latter possibility.

An unexpected finding among our sample set was an isolate from Uganda that encoded an amino acid change in the *pmcytb* gene only previously reported from a wild-caught chimpanzee in Sierra Leone. This provides some support for the hypothesis that African malaria parasites of all species shuttle between great ape and human hosts [29], as recent data for both *P*. *falciparum* and *P*. *vivax* suggest [30,31]. This form of *pmcytb* could be of human origin, existing as a minor variant across *P*. *malariae* populations among *Homo sapiens* in Africa, which happened to be transferred to *Pan troglodytes verus* in Cote d’Ivoire. Alternatively, there may be genetically diverse *P*. *malariae* across the wild ape populations of central and western Africa, which provide a reservoir of new genetic variants for human-focussed transmission cycles [27]. Further sampling and sequencing in both hosts is needed to explore these possibilities.

We find support in our data for the notion that *P*. *malariae* is a truly relapsing parasite species, and has a quiescent liver-stage form analogous to the well-described hypnozoites of *P*. *vivax* and the simian parasite *P*. *cynomolgi*. This hypothesis could be tested experimentally, as Dembele *et al* [32] have recently been able to infect hepatocytes of *Macaca fascicularis* with sporozoites of *Plasmodium cynomolgi* and directly demonstrate persistence and later activation of hypnozoites. Infection of human hepatocytes with *P*. *malariae* sporozoites should be technically feasible. Our conclusion is in contradiction to the findings of Ciuca *et al*. [5], who administered a Romanian strain of *P*. *malariae* to three volunteers by sporozoite inoculation together with a single dose of 50mg pyrimethamine. Over 8 to 18 months’ follow-up, none of the volunteers developed a blood-stage *P*. *malariae* infection. Similarly, six volunteers with experimentally-induced blood-stage parasitaemia with the same Romanian *P*. *malariae* isolate were successfully treated with various antimalarial regimens. No relapses occurred in any of these volunteers over 15 months’ follow-up. These authors conclude the following: “Ces constatations imposent la conclusion logique que *P*. *malariae* n’aurait qu’une phase tissulaire préérythrocytaires unique << la schizogonie exoérythrocytaire primaire>>.” (There is only a single hepatic shizogony cycle in each infection.) [5] Coatneyi *et al* comment on these findings of Ciuca *et al* thus: “In this connection, we also have observed no relapse activity after adequate blood schizonticidal therapy in our volunteers exposed to infection with a Nigerian strain of *P*. *malariae*. It seems safe to say that, in this respect, *P*. *malariae* resembles *P*. *falciparum* more than it does the human tertian malarias which do relapse.” [33]

There are several tentative explanations that might help us to reconcile our findings with the earlier evidence from experimentally induced infections that *P*. *malariae* does not relapse. Firstly, the initiation of a secondary hepatic cycle may be a rare event in *P*. *malariae* and thus a handful of volunteers may not have been a large enough sample to detect relapse events. Secondly, follow-up of less than 2 years may have missed late events in these individuals, as *P*. *malariae* is without doubt the human parasite with the greatest within-host longevity. Thirdly, the putative hypnozoite stage of *P*. *malariae* may not be identical to that of *P*. *vivax* in its mechanism of arrest; if metabolism halts at a later stage of intra-hepatocytic development, these forms may not be completely insensitive to full therapeutic doses of pyrimethamine or other antimalarials (although would appear able to escape low-dose chemo-prophylaxis). Finally, some “strains” of *P*. *malariae* may have lost the ability to form hypnozoite-like stages in the host liver, and thus the single Romanian parasite line of Ciuca *et al*. [5], and the single Nigerian line described by Coatneyi *et al*. [33] could have been non-relapsing types. Further studies are required to tease apart these various alternatives, and the recent development of appropriate humanised mouse models for liver-stage studies offers an exciting new strategy for pursuit of this question [34].

### Conclusions

Analysis of delay to onset of symptoms in a large series of imported quartan malaria cases in three non-endemic countries, and elucidation of the *pmcytb* sequences of *P*. *malariae* isolates from a subset of users of atovaquone-proguanil chemoprophylaxis, lead us to conclude that *P*. *malariae* may be able to form hypnozites, or an analogous liver-stage form. As Garnham has observed: “… true long term relapses of sporozoite induced infections seem more likely to be due to a secondary exoerythrocytic cycle in the liver, known to occur in other species, than to a persistent low-grade parasitaemia.” (P. 269) [35]. Whereas Garnham subsequently changed his view [36], our data and the work of others [11; references therein] support re-evaluation of the possibility that *P*. *malariae* is a relapsing parasite.

## References

[1] Sutherland CJ, Tanomsing N, Nolder D, Oguike M, Jennison C, Pukrittayakamee S, et al. Two nonrecombining sympatric forms of the human malaria parasite Plasmodium ovale occur globally. J Infect Dis, 2010; 201: 1544–1550. 10.1086/652240 20380562

[2] World Health Organisation. World Malaria Report: 2013. http://www.who.int/malaria/publications/world_malaria_report_2014 (accessed 2^nd^ July 2015.

[3] Public Health England: https://www.gov.uk/government/publications/imported-malaria-in-the-uk-statistics (accessed 2^nd^ July 2015).

[4] Smith AD, Bradley DJ, Smith V, Blaze M, Behrens RH, Chiodini PL, Whitty CJ. Imported malaria and high risk groups: observational study using UK surveillance data 1987–2006. Br Med J, 2008; 337: a120. 18599471 10.1136/bmj.a120 PMC2453297

[5] Ciuca M, Lupasco G, Negulici E, Constantinesco P. [Research on the experimental transmission of Plasmodium malariae to man]. Arch Roum Pathol Exp Microbiol, 1964; 23: 763–776. 5318792

[6] Collins WE, Jeffery GM. Plasmodium malariae: parasite and disease. Clin Microbiol Rev, 2007; 20: 579–592. 17934075 10.1128/CMR.00027-07 PMC2176047

[7] Mombo-Ngoma G, Kleine C, Basra A, Würbel H, Diop DA, Capan M, et al Prospective evaluation of artemether-lumefantrine for the treatment of non-falciparum and mixed-species malaria in Gabon. Malar J. 2012; 11: 120 10.1186/1475-2875-11-120 22515681 PMC3393621

[8] Dinko B, Oguike MC, Larbi JA, Bousema T, Sutherland CJ. Persistent detection of Plasmodium falciparum, P. malariae, P. ovale curtisi and P. ovale wallikeri after ACT treatment of asymptomatic Ghanaian school-children. Int J Parasitol DDR, 2013; 3: 45–50. 10.1016/j.ijpddr.2013.01.001 PMC3862415 24533292

[9] Betson M, Sousa-Figueiredo JC, Atuhaire A, Arinaitwe M, Adriko M, Mwesigwa G, *et el*. Detection of persistent Plasmodium spp. infections in Ugandan children after artemether-lumefantrine treatment. Parasitology 2014; 141: 1880–1890. 10.1017/S003118201400033X 24837880 PMC4255323

[10] Hong YJ, Yang SY, Lee K, Kim TS, Kim HB, Park KU, et al A case of imported Plasmodium malariae malaria. Ann Lab Med, 2012; 32: 229–233. 10.3343/alm.2012.32.3.229 22563561 PMC3339306

[11] Franken G, Müller-Stöver I, Holtfreter MC, Walter S, Mehlhorn H, Labisch A, et al Why do Plasmodium malariae infections sometimes occur in spite of previous antimalarial medication? Parasitol Res, 2012; 111: 943–946. 10.1007/s00436-012-2851-8 22350675

[12] Vinetz JM, Li J, McCutchan TF, Kaslow DC. Plasmodium malariae infection in an asymptomatic 74-year-old Greek woman with splenomegaly. N Engl J Med, 1998; 338: 367–371. 9449730 10.1056/NEJM199802053380605

[13] Chadee DD, Tilluckdharry CC, Maharaj P, Sinanan C. Reactivation of Plasmodium malariae infection in a Trinidadian man after neurosurgery. N Engl J Med, 2000; 342: 1924 10877649 10.1056/NEJM200006223422520

[14] Hedelius R1, Fletcher JJ, Glass WF II, Susanti AI, Maguire JD. Nephrotic syndrome and unrecognized Plasmodium malariae infection in a US Navy sailor 14 years after departing Nigeria. J Travel Med. 2011; 18: 288–291. 10.1111/j.1708-8305.2011.00526.x 21722243

[15] Krotoski WA, Krotoski DM, Garnham PC, Bray RS, Killick-Kendrick R, Draper CC, et al Relapses in primate malaria: discovery of two populations of exoerythrocytic stages. Br Med J, 1980; 280: 153–154. 6766771 10.1136/bmj.280.6208.153-a PMC1600318

[16] Krotoski WA, Garnham PC, Cogswell FB, Collins WE, Bray RS, Gwasz RW, et al Observations on early and late post-sporozoite tissue stages in primate malaria. IV. Pre-erythrocytic schizonts and/or hypnozoites of Chesson and North Korean strains of Plasmodium vivax in the chimpanzee. Am J Trop Med Hyg, 1986; 35: 263–274. 3513645 10.4269/ajtmh.1986.35.263

[17] Chiodini PL, Field VK, Hill DR, Whitty CJM, Lalloo DG. Guidelines for malaria prevention in travellers from the United Kingdom London, Public Health England, 7 2013.

[18] Looareesuwan S, Wilairatana P, Glanarongran R, Indravijit KA, Supeeranontha L, Chinnapha S, et al Atovaquone and proguanil hydrochloride followed by primaquine for treatment of Plasmodium vivax malaria in Thailand. Transactions Roy Soc Trop Med Hyg, 1999; 93: 637–640. 10.1016/s0035-9203(99)90079-2 10717754

[19] Radloff PD, Philipps J, Hutchinson D, Kremsner PG. Atovaquone plus proguanil is an effective treatment for Plasmodium ovale and P. malariae malaria. Trans Roy Soc Trop Med Hyg. 1996; 90: 682 9015517 10.1016/s0035-9203(96)90435-6

[20] Nolder D, Oguike MC Maxwell-Scott H, Niyazi HA, Smith V, Chiodini PL, Sutherland CJ. Malaria in British travellers: Plasmodium ovale wallikeri and P. ovale curtisi differ significantly in the duration of latency. BMJ Open, 2013; 3: e002711 10.1136/bmjopen-2013-002711 23793668 PMC3657643

[21] Fivelman QL, Butcher GA, Adagu IS, Warhurst DC, Pasvol G. Malarone treatment failure and in vitro confirmation of resistance of Plasmodium falciparum isolate from Lagos, Nigeria. Malaria J, 2002; 1:1. 10.1186/1475-2875-1-1 PMC111499 12057021

[22] Musset L, Bouchaud O, Matheron S, Massias L, Le Bras J. Clinical atovaquone-proguanil resistance of Plasmodium falciparum associated with cytochrome b codon 268 mutations. Microbes and infection, 2006; 8: 2599–2604. 16962361 10.1016/j.micinf.2006.07.011

[23] Sutherland CJ, Laundy M, Price N, Burke M, Fivelman QL, Pasvol G et al Mutations in the Plasmodium falciparum cytochrome b gene are associated with delayed parasite recrudescence in malaria patients treated with atovaquone-proguanil. Malaria J, 2008; 7: 240. 10.1186/1475-2875-7-240 PMC2640403 19021900

[24] Kofoed K, Petersen E. The efficacy of chemoprophylaxis against malaria with chloroquine plus proguanil, mefloquine, and atovaquone plus proguanil in travelers from Denmark. J Travel Med, 2003; 10: 150–154. 12757688 10.2310/7060.2003.35746

[25] Povinelli L, Monson TA, Fox BC, Parise ME, Morrisey JM, Vaidya AB. Plasmodium vivax malaria in spite of atovaquone/proguanil (malarone) prophylaxis. J Travel Med, 2003; 10: 353–355. 14642204 10.2310/7060.2003.9318

[26] Schwartz E, Parise M, Kozarsky P, Cetron M. Delayed onset of malaria—implications for chemoprophylaxis in travelers. N Engl J Med, 2003; 349: 1510–1516. 14561793 10.1056/NEJMoa021592

[27] Kaiser M, Löwa A, Ulrich M, Ellerbrok H, Goffe AS, Blasse A et al. Wild chimpanzees infected with 5 Plasmodium species. Emerg Infect Dis, 2010; 16: 1956–1959. 10.3201/eid1612.100424 21122230 PMC3294549

[28] Srivastava IK, Morrisey JM, Darrouzet E, Daldal F, Vaidya AB. Resistance mutations reveal the atovaquone-binding domain of cytochrome b in malaria parasites. Mol Microbiol, 1999; 33: 704–711. 10447880 10.1046/j.1365-2958.1999.01515.x

[29] Sutherland CJ, Polley SD. 2011 “Genomic insights into the past, current and future evolution of human parasites of the genus Plasmodium” In: Genetics and Evolution of Infectious Diseases Tibayrenc M. (Ed.). Elsevier, London, 2011: 607–635. ISBN: 978-0-12-384890-1.

[30] Liu W, Li Y, Learn GH, Rudicell RS, Robertson JD, Keele BF, Ndjango JB et al Origin of the human malaria parasite Plasmodium falciparum in gorillas. Nature, 2010; 467: 420–425. 10.1038/nature09442 20864995 PMC2997044

[31] Liu W, Li Y, Shaw KS, Learn GH, Plenderleith LJ, Malenke JA et al. African origin of the malaria parasite Plasmodium vivax. Nat Commun. 2014; 5: 3346 10.1038/ncomms4346 24557500 PMC4089193

[32] Dembélé L, Franetich JF, Lorthiois A, Gego A, Zeeman AM, Kocken CH et al Persistence and activation of malaria hypnozoites in long-term primary hepatocyte cultures. Nat Med. 2014; 20: 307–312. 10.1038/nm.3461 24509527

[33] Coatney GR, Collins WE, Warren McW, Contacos PG. 1971. The primate malarias [original book published 1971]. CD-ROM; CDC Division of Parasitic Disease, 2003. Version 1.0. Atlanta, GA.

[34] Soulard V, Bosson-Vanga H, Lorthiois A, Roucher C, Franetich JF, Zanghi G. et al 2015. *Plasmodium falciparum* full life cycle and *Plasmodium ovale* liver stages in humanized mice. Nat Commun. 2015 6: 7690 10.1038/ncomms8690 26205537 PMC4525212

[35] Garnham PCC. 1966 Malaria parasites and other haemosporidia Blackwell, Oxford. 1114 pages.

[36] Garnham PCC. The myth of quartan malaria. Trans Roy Soc Trop Med Hyg. 1981; 75: 616–617. 7324148 10.1016/0035-9203(81)90229-7

